# Long non-coding RNA (LncRNA) MRPL23-AS1 promotes tumor progression and carcinogenesis in osteosarcoma by activating Wnt/β-catenin signaling via inhibiting microRNA miR-30b and upregulating myosin heavy chain 9 (MYH9)

**DOI:** 10.1080/21655979.2020.1863014

**Published:** 2020-12-28

**Authors:** Hanwen Zhang, Shuya Liu, Lian Tang, Jianhua Ge, Xiaobo Lu

**Affiliations:** a Department of Orthopaedics, The Affiliated Hospital of Southwest Medical University, Luzhou City, P.R. China; b Department of Oncology, The Affiliated Hospital of Southwest Medical University, Luzhou City, P.R. China

**Keywords:** LncRNA, MRPL23-AS1, osteosarcoma, Wnt/β-catenin signaling, prognosis

## Abstract

Long non-coding RNA (LncRNA) contributes to the occurrence and development of osteosarcoma (OS), although the underlying mechanism is not clear. In the present study, we showed that lncRNA MRPL23-AS1 was remarkably increased in OS tissues and cell lines. Stable knockdown of MRPL23-AS1 evidently attenuated cell viability and invasive ability, meanwhile inhibited *in vivo* tumor growth and dissemination. In terms of mechanism, luciferase reporter, RNA pull-down and fluorescence in situ hybridization (FISH) assays showed that MRPL23-AS1 competitively interacted with miR-30b, increasing myosin heavy chain 9 (MYH9) expression, a trans- activator of β-catenin, resulting in the activation of Wnt/β-catenin pathway, thereby promoting OS tumorigenesis and metastasis. Importantly, high MRPL23-AS1 was positively correlated with MYH9, while conversely correlated with miR-30b, suggesting that the regulatory axis of MRPL23-AS1/miR-30b/MYH9 does exist in OS. Clinically, OS patients with high MRPL23-AS1 had larger tumor size, higher stage and easier metastasis than those with low MRPL23-AS1, moreover, MRPL23-AS1 was identified as an adverse prognostic factor for OS survival. In conclusion, our results show that MRPL23-AS1 is a key oncogenic lncRNA in OS, targeting of MRPL23-AS1 may be a promising treatment for OS patients.

## GRAPHICAL ABSTRACT

**Figure:**
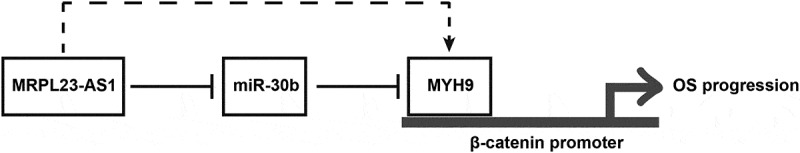

## Introduction

Osteosarcoma (OS) is a malignant tumor in which tumor cells can directly form tumor-like bone tissue or bone tissue, accounting for about 20 ~ 34% of all primary malignant bone tumors [1]. It is characterized by high degree of malignancy, rapid development and metastasis in early stage [2]. The current treatment is a combination of multiple chemotherapy and surgery, but it is still largely inadequate because the survival time has not been improved in recent decades [1]. Therefore, it is important and urgent to explore the mechanisms of OS pathogenesis and metastasis, which may lead to new directions for clinical treatment.

DNA, RNA and protein are three important biological macromolecules, which are the molecular basis of life phenomena. RNA is not only involved in protein translation, but also involved in gene expression regulation, it is a kind of biological macromolecule with wide functions [3]. Long non-coding RNA (LncRNA) is a class of endogenous RNA molecule with a length of more than 200 nucleotides, which plays an important role in many life activities [4], such as dose compensation effect, epigenetic regulation, cell cycle and differentiation regulation [5]. LncRNA functions in a variety of modes of action, the most well-known is being ‘microRNA (miRNA) molecular sponge ’ [6]. In this functional model, lncRNA competitively binds miRNA to reduce the interaction between miRNA and its target genes, resulting in the upregulation of targets [6]. This indirect regulatory axis is also known as competing endogenous RNA (ceRNA) network [7]. Emerging evidence suggests that the lncRNA/miRNA/mRNA axis is frequently abnormally regulated in human diseases, especially in cancer [8]. For instance, BCYRN1 was reported as a tumor suppressor in glioma, it could sponge miR-619-5p and elevate CUEDC2, inactivating PTEN/AKT/p21 pathway [9]; LINC01134 was significantly increased in hepatocellular carcinoma and promoted cell proliferation and angiogenesis via absorbing miR-4784 and upregulating SSRP1 [10].

Recently, a novel lncRNA, MRPL23-AS1, was proposed as a driver of cystic carcinoma lung metastasis, it was mainly located in the nucleus, and could increase the binding of EZH2 and H3K27me3 on the E-cadherin promoter region, leading to the transcriptional silencing of E-cadherin [11]. However, its role in OS remains unknown. In the present study, we aimed to explore the expression, function and clinical significance of MRPL23-AS1 in OS, and further reveal the underlying regulatory mechanism of MRPL23-AS1 in promoting OS progression.

## Materials and methods

### Tissue samples

OS and adjacent normal tissue samples from 89 OS patients who underwent surgical resection at The Affiliated Hospital of Southwest Medical University were collected and immediately stored in liquid nitrogen. The tissues were diagnosed as OS by two independent and experienced pathologists. The clinicopathological information of all enrolled patients was obtained, and patients who had received chemoradiotherapy before surgery were excluded. We obtained written informed consent from each patient, and this study was approved by the Ethics Committee of The Affiliated Hospital of Southwest Medical University.

### Cell lines and transfection

One normal hFOB1.19 cells and four OS cell lines including HOS, 143B, U2OS and MG63 were obtained from ATCC. DMEM or RPMI-1640 medium and 10% fetal bovine serum were used to culture above cells. miR-30b mimics (5`-CUGGGAGGUGGAUGUUUACUUC-3`) and myosin heavy chain 9 (MYH9) siRNA (5`-GGAAGAAGGUGAAGGUGAA-3`) were purchased from RiboBio (Guangzhou, China), they were transfected into OS cells using Lipofectamine™ 2000 as per manufacturer’s protocols (Invitrogen, Carlsbad, CA, USA).

### Construction of MRPL23-AS1-depleted or -overexpressed OS cells

To silence MRPL23-AS1, three shRNAs targeting MRPL23-AS1 were designed and synthesized (shRNA#1: 5`-CACAGCGCUGAGAAAGUAA-3`; shRNA#2: 5`-GGAAAUACAGCCCUACUUC-3`; shRNA#3: 5`-GCAAAUUCUCACCUCCAGA-3`;), followed by insertion into pGLVU6/RFP/Puro lentivirus vector (GenePharma, Shanghai, China). Next, the cells were grown and selected in growth media containing 2 μg/mL puromycin. The puromycin-resistant colonies were then selected, pooled and cultured for further experiments under puromycin-selection conditions. To overexpress MRPL23-AS1, the full-length of MRPL23-AS1 was synthesized, followed by insertion into pcDNA 3.0 vector (Invitrogen), then, the vector was transfected into OS cells using Lipofectamine™ 2000 reagent.

### qRT-PCR analysis

Total RNA was extracted from OS tissues and cell lines using Trizol reagent (Invitrogen), followed by reverse transcription into cDNA using PrimeScript™ Reverse Transcriptase kit (Takara, Shiga, Japan), qPCR was performed with a ChamQTM SYBR^@^ qPCR Master Mix (Vazyme Biotech, Nanjing, China). All reactions were run in triplicate on the CFX96^TM^ Touch Real-Time PCR System (Bio-Rad, California, USA). GAPDH or U6 were applied as endogenous control. The relative expression was calculated using the comparative Ct (2^−ΔΔCT^) method.

### Determination of MRPL23-AS1 location

The Cytoplasmic & Nuclear RNA Purification Kit was purchased from Norgen Biotek (Thorold, ON, Canada) using for rapidly isolation and purification of cytoplasmic and nuclear RNA from hFOB1.19 and U2OS cells. GAPDH and U6 were used as endogenous controls for cytoplasmic and nuclear fragments, respectively. Besides, FISH assay was performed by using commercialized FISH kit (GenePharma) according to the supplier’s instructions.

### CCK-8 and transwell assays

OS cells were plated into 96-well plates at appropriate density, followed by addition into 10 μL CCK-8 solution (Dojindo, Kumamoto, Japan) at 24 h, 48 h and 72 h. After incubation at 37°C for 2 h, the plates were put into a microplate reader, and the absorbance value of each well was detected. For cell invasion assay, the Transwell chamber coated with matrigel (Corning, NY, USA) was used. After passing through the matrigel, the cells were fixed with methanol, and stained with crystal violet.

### In vivo *experiments*

The animal protocols of this study were approved by the Institutional Animal Care and Use Committee (IACUC) of The Affiliated Hospital of Southwest Medical University. For the subcutaneous tumorigenesis experiment, a total of 1 × 10^7^ OS cells with or without MRPL23-AS1 knockdown were injected subcutaneously into male athymic BALB/c nude mice at 4 to 5 weeks of age. After four weeks, all mice were sacrificed and the tumor tissues were weighed. For the experimental lung metastasis model, 1 × 10^6^ OS cells were injected into nude mice through the caudal vein. After six weeks, mice were abdominally injected with D-luciferin (200 mg/kg), anesthetized and imaged with Xenogen IVIS Lumina system (Caliper Life Sciences, Hopkinton, MA, USA).

### RNA pull-down assay

The MRPL23-AS1 were transcribed *in vitro* using a MEGA script™ T7 Transcription Kit (Invitrogen) and biotinylated using Pierce™ Biotin 3' End DNA Labeling Kit (Thermo Fisher Scientific, Waltham, MA, USA) based on the standard protocols. Then, the MRPL23-AS1 probe was incubated with OS cell lysate overnight at 4°C. The next day, cell lysate was added with streptavidin-conjugated magnetic beads (Invitrogen), followed by incubation at 4°C for 1 h. The mixtures were washed five times through centrifugation, and the affinity-purified RNA was extracted by TRIzol reagent, followed by qRT-PCR analysis of miRNA enrichment.

### Luciferase reporter gene assay

OS cells (1 × 10^5^) were seeded into 6-well plates, grown overnight, and then co-transfected with miR-30b mimics and MRPL23-AS1 or MYH9 3`-UTR pmiR-GLO luciferase construct using Lipofectamine™ 2000 for 24 h. Furthermore, to assess the MRPL23-AS1-mediated CTNNB1 transcription in activation of the Wnt/β-catenin signaling pathway, the TOP/FOP-flash assay was used by transfecting OS cells with TOP-flash (the TCF reporter plasmid; Merck Millipore, Schwalbach, Germany) or FOP-flash (plasmid carrying the mutant TCF binding sites; Merck-Millipore) using Lipofectamine™ 2000 for 24 h, while the renilla luciferase reporter plasmid pR-TK (Promega, Madison, WI, USA) was used as the control. The luciferase activity was measured by using the Promega Dual-Glo® luciferase assay system (Promega) as per the supplier’s protocols.

### Western blot

OS cell lysates were prepared using the Mammalian Protein Extraction Kit (CWBIO, Beijing, China), the protein concentration was tested using the Bicinchoninic Acid (BCA) Protein Assay Kit (CWBIO). Then, the protein was separated on 10% sodium dodecyl sulfate-polyacrylamide gel electrophoresis (SDS-PAGE) gels and transferred onto nitrocellulose membranes (Bio-Rad, Hercules, CA, USA). After blocking with 5% nonfat milk powder, the membranes were incubated with primary antibody including anti-MYH9 (#sc-98978, Santa Cruz Biotechnology, Dallas, Texas, USA), anti-β-catenin (#610154, BD Biosciences, San Jose, CA, USA), anti-c-Myc (#ab40798, Abcam, Cambridge, UK), anti-cyclin D1 (#ab226977, Abcam), anti-GAPDH (#sc-32233, Santa Cruz Biotechnology) and anti-Mouse/Rabbit IgG H&L (HRP) secondary antibody. The positive protein signal was visualized with the Immobilon-Western HRP kit (Millipore) and exposed to X-ray films.

### Statistical analysis

The *in vitro* functional results were shown as the mean±SD of at least three independent experiments carried out in triplicate. The correlations between MRPL23-AS1 and clinicopathological characteristics of OS patients were determined by Chi-square test. Differences between groups were compared as appropriate using Student’s t test (two groups) or Analysis of Variance (ANOVA) (three or more groups) with Tukey honestly significant difference (HSD) post hoc test. The follow-up interval was 6 months, and Kaplan-Meier method was used to plot survival curve, which was analyzed by Log-rank test. Statistical analysis and figure generation were performed by GraphPad Prism 8.0 software (Graph-Pad, San Diego, CA, USA). *P* value < 0.05 was considered statistically significant. *p < 0.05, **p < 0.01, ***p < 0.001.

## Results

Here, we aimed to explored the role of MRPL23-AS1 in OS. We conducted a series of *in vitro* and *in vivo* assays, and found that MRPL23-AS1 promoted OS progression via regulating the miR-30b/MYH9/Wnt/β-catenin axis. Therefore, our data for the first investigated the functional and clinical roles of MRPL23-AS1 in OS, providing new insights into the pathogenesis of OS.

### MRPL23-AS1 is frequently overexpressed in OS tissues and cell lines

**Figure 1.**
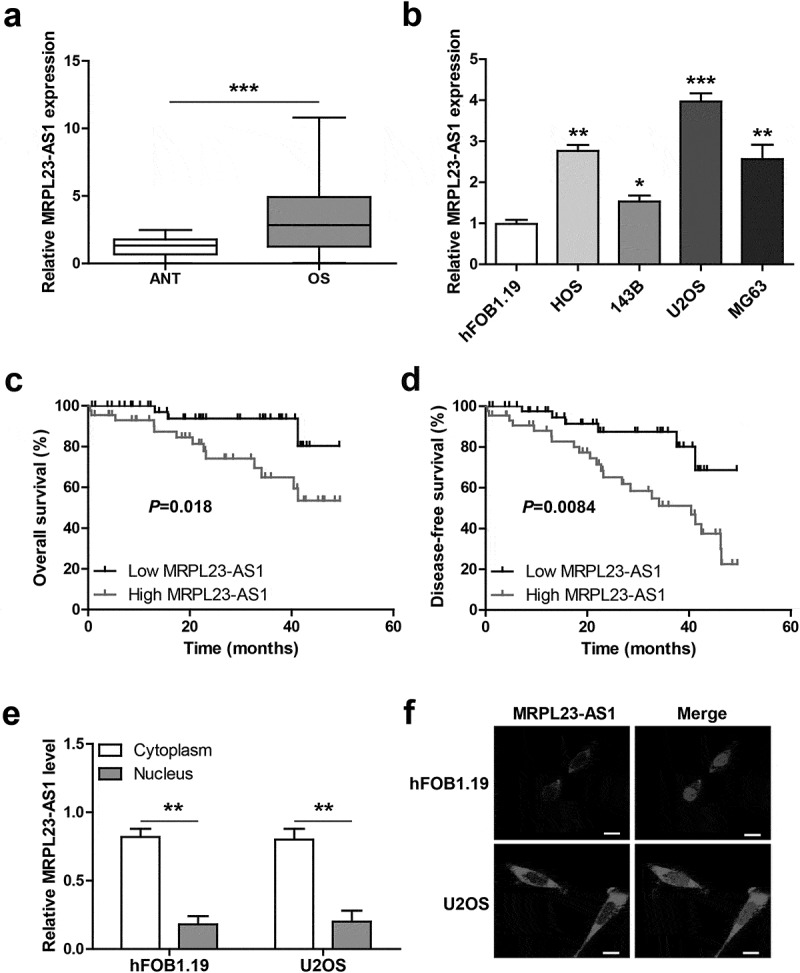
MRPL23-AS1 is significantly increased in OS. (a,b) qRT-PCR analysis of MRPL23-AS1 in OS tissues and cell lines. (c,d) The overall and disease-free survival curves of OS patients with low or high MRPL23-AS1 expression. (e,f) qRT-PCR and FISH assays detecting the location of MRPL23-AS1 in OS cells. *p < 0.05, **p < 0.01, ***p < 0.001

We first detected the expression of MRPL23-AS1 in OS tissues, the results showed that as compared to adjacent normal tissues, MRPL23-AS1 was significantly increased in OS tissues (Figure 1(a)). Likewise, MRPL23-AS1 expression in OS cell lines was also higher than that in normal hFOB1.19 cells (Figure 1(b)). Then, we analyzed the correlations between MRPL23-AS1 and clinicopathological characteristics. As shown in Table 1, high MRPL23-AS1 was positively correlated with larger tumor size, later clinical stage and metastasis, while was not correlated with gender, age and differentiation grade. Importantly, OS patients with high MRPL23-AS1 expression had shorter overall and disease-free survival time than those with low MRPL23-AS1 expression (Figure 1(c,d)). Next, we tested the subcellular localization of MRPL23-AS1 by conducting qRT-PCR and FISH assays, the results showed that MRPL23-AS1 was preferentially located in the cytoplasm (Figure 1(e,f)).

**Table 1.** The correlations between MRPL23-AS1 expression and clinicopathological characteristics in 89 OS patients

Parameters	All cases	MRPL23-AS1 expression		*P* value
		Low	High	
Gender				
Male	52	24	28	0.324
Female	37	21	16	
Age (years)				
≤ 20	55	25	30	0.220
> 20	34	20	14	
Tumor size				
≤ 8	60	36	24	0.010
> 8	29	9	20	
Metastasis				
No	40	29	11	0.000
Yes	49	16	33	
TNM stage				
I–II	51	33	18	0.002
III–IV	38	12	26	
Differentiation				
Well/moderate	56	31	25	0.239
Poor	33	14	19

### *MRPL23-AS1 promotes OS cell growth and metastasis both* in vitro *and* in vivo

**Figure 2.**
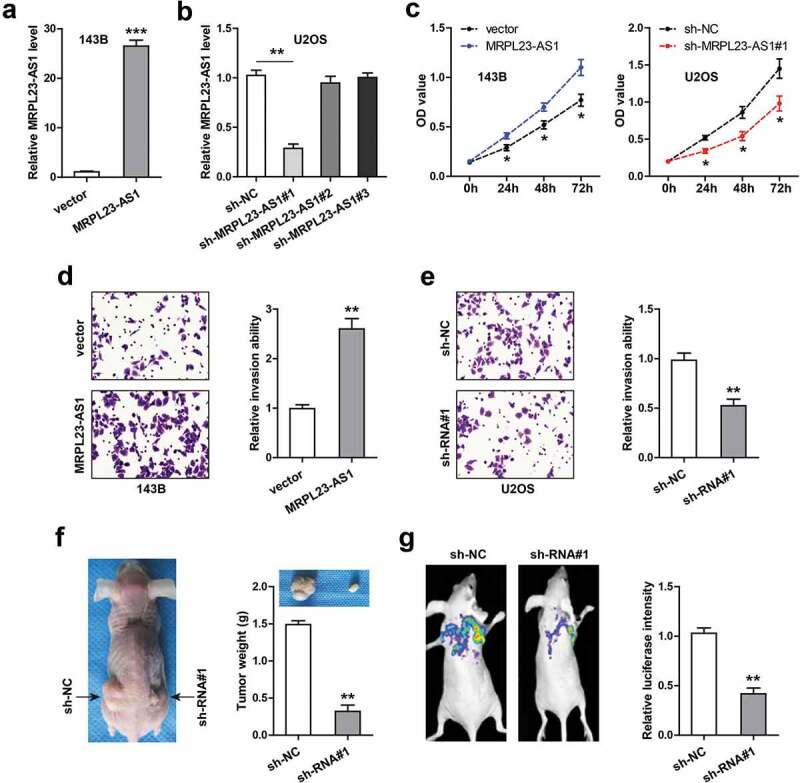
MRPL23-AS1 promotes OS cell proliferation and invasion *in vitro* and *in vivo*. (a,b) qRT-PCR verifying the construction of stable MRPL23-AS1-overexpressing 143B cells and MRPL23-AS1-depletd U2OS cells. (c) CCK-8 assay detecting the viability of OS cells after alteration of MRPL23-AS1 level. (d,e) Transwell assay analyzing cell migration and invasion after MRPL23-AS1 knockdown or overexpression. (f) The representative image of nude mice bearing control or MRPL23-AS1-silenced U2OS cells. (g) Bioluminescence imaging showing the effect of MRPL23-AS1 knockdown on *in vivo* lung metastasis of U2OS cells. *p < 0.05, **p < 0.01, ***p < 0.001

To explore the function of MRPL23-AS1 in OS, we chose 143B cells with relatively low MRPL23-AS1 expression to overexpress MRPL23-AS1 (Figure 2(a)), meanwhile chose U2OS cells with relatively high MRPL23-AS1 expression to silence MRPL23-AS1 (Figure 2(b)). Specifically, for MRPL23-AS1 knockdown, we designed three shRNAs against MRPL23-AS1, and the qRT-PCR results showed that only sh-MRPL23-AS1#1 could effectively knock down MRPL23-AS1 (Figure 2(b)). The CCK-8 assays showed that MRPL23-AS1 overexpression significantly increased cell viability, while MRPL23-AS1 depletion resulted in an opposite trend (Figure 2(c)). Similarly, the invasive ability of 143B cells was remarkably enhanced after ectopic expression of MRPL23-AS1 (Figure 2(d)), and that of U2OS cells was significantly weakened after silencing of MRPL23-AS1 (Figure 2(e)). Further, we conducted the xenograft tumor experiment, the results displayed that the tumor size in MRPL23-AS1-depleted group was evidently smaller than that in control group (Figure 2(f)). And less lung metastasis nodules were observed in MRPL23-AS1-depleted group in comparison to control group (Figure 2(g)).

### MRPL23-AS1 acts as a sponge of miR-30b

**Figure 3.**
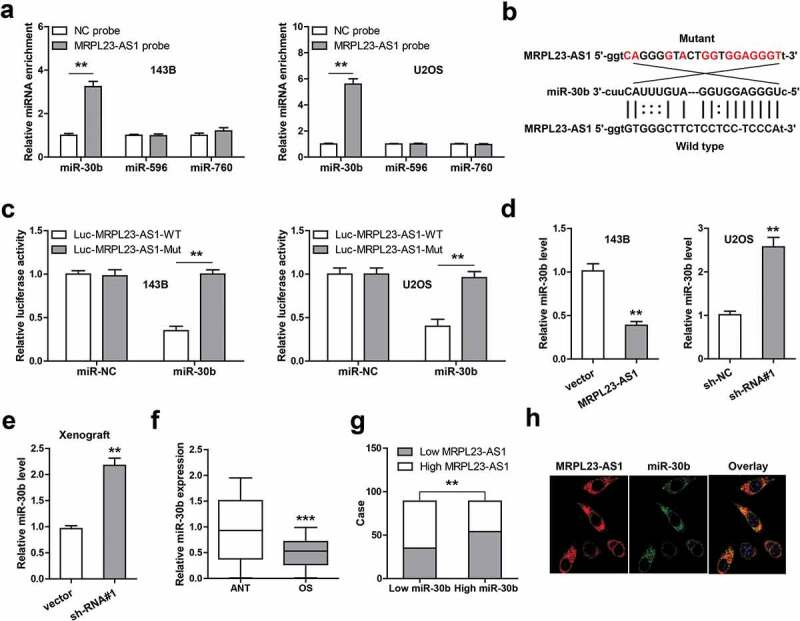
MRPL23-AS1 serves as a sponge of miR-30b in OS cells. (a) RNA pull-down assay using biotin-labeled MRPL23-AS1 probe, followed by qRT-PCR analysis of enrichment of miR-30b, miR-596 and miR-760. (b) The wild-type or mutant binding site between MRPL23-AS1 and miR-30b. (c) Luciferase reporter gene assay detecting the luciferase activity of wild-type or mutant MRPL23-AS1 vector after miR-30b overexpression. (d) qRT-PCR analysis of miR-30b expression after overexpression or silencing of MRPL23-AS1. (e) qRT-PCR analysis of miR-30b expression in control or MRPL23-AS1-silenced xenograft tumor tissues. (f) qRT-PCR analysis of miR-30b expression in OS and matched normal tissues. (g) The correlation between MRPL23-AS1 and miR-30b in OS tissues. (h) FISH assay showing the co-location between MRPL23-AS1 and miR-30b in OS cells. **p < 0.01, ***p < 0.001

In the light of the cytoplasmic location of MRPL23-AS1 in OS cells, we then inferred that MRPL23-AS1 may be a ceRNA. Through integrating the predicted results of RegRNA2, miRCode and lncRNASNP2 software, we found that three miRNAs might bind to MRPL23-AS1, namely miR-30b, miR-596 and miR-760. The RNA pull-down assays showed that only miR-30b could be effectively enriched by MRPL23-AS1 probe in both 143B and U2OS cells (Figure 3(a)). We then mutated the binding site between MRPL23-AS1 and miR-30b (Figure 3(b)), and conducted the luciferase reporter gene assay. As shown in Figure 3(c), miR-30b overexpression significantly reduced luciferase activity of wild-type vector, while this effect was disappeared after mutation of the binding site. Furthermore, MRPL23-AS1 overexpression in 143B cells decreased miR-30b expression, whereas MRPL23-AS1 knockdown in U2OS cells led to an opposite result (Figure 3(d)). And miR-30b was significantly upregulated in MRPL23-AS1-depleted xenograft tumor tissue (Figure 3(e)). Importantly, low miR-30b was detected in OS tissues as compared to normal tissues (Figure 3(f)), and its level was negatively correlated with MRPL23-AS1 level (Figure 3(g)). The FISH results showed that MRPL23-AS1 and miR-30b was co-located in the cytoplasm (Figure 3(h)).

### MRPL23-AS1 regulates the miR-30b/MYH9/Wnt/β-catenin axis

**Figure 4.**
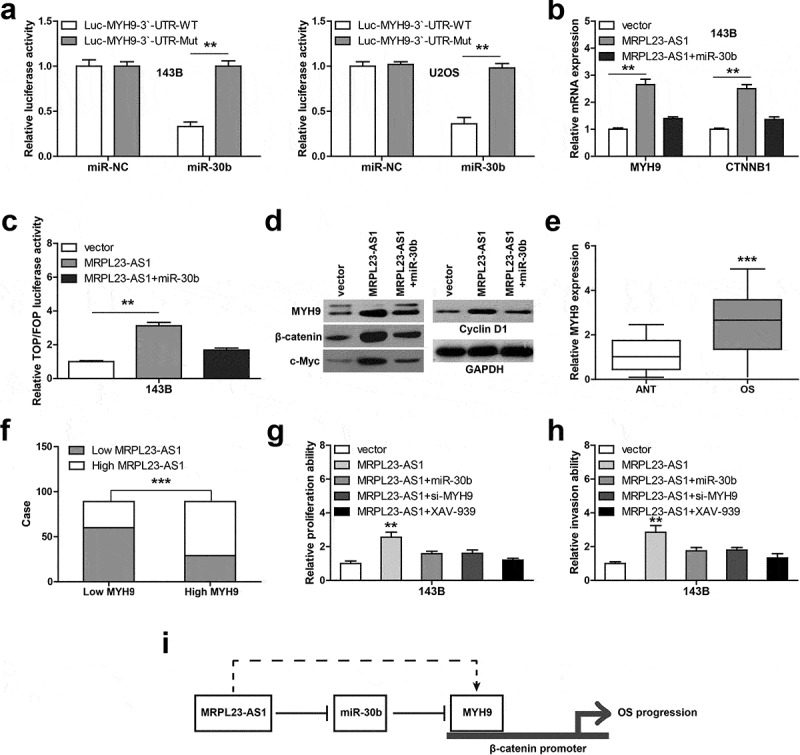
MRPL23-AS1 regulates the miR-30b/MYH9/Wnt/β-catenin axis in OS cells. (a) Luciferase reporter gene assay detecting the luciferase activity of wild-type or mutant MYH9 3`-UTR vector after miR-30b overexpression. (b) qRT-PCR analysis of MYH9 and β-catenin mRNA levels in MRPL23-AS1-overexpressing 143B cells after miR-30b overexpression. (c) Luciferase reporter gene assay detecting the luciferase activity of TOP/FOP-Flash reporter in MRPL23-AS1-overexpressing 143B cells after miR-30b overexpression. (d) Western blotting analyzing protein levels of MYH9, β-catenin, c-Myc and Cyclin D1, GAPDH was used as the loading control. (e) qRT-PCR analysis of MYH9 expression in OS and matched normal tissues. (f) The correlation between MRPL23-AS1 and MYH9 in OS tissues. (g,h) The relative proliferation and invasion of MRPL23-AS1-overexpressing 143B cells after transfection with miR-30b mimics, MYH9 siRNA or treatment with XAV-939. **p < 0.01, **p < 0.001. (i) The diagrammatic sketch showing MRPL23-AS1 promoting OS progression via sponging miR-30b and elevating MYH9, ultimately activating oncogenic Wnt/β-catenin pathway. **p < 0.01, ***p < 0.001

By analyzing the online tool miRWalk, we found that MYH9 might be the downstream target of miR-30b. We then conducted the luciferase reporter assay, the results showed that miR-30b overexpression resulted in a significant decrease of the luciferase activity of the wild-type MYH9 vector, whereas did not affect that of the mutant one (Figure 4(a)). The RNA pull-down assay showed that miR-30b abundantly bound to MYH9 3`-UTR region (Figure S1). Furthermore, MRPL23-AS1 overexpression remarkably increased the mRNA levels of MYH9 and its target β-catenin, while these effects were partially blocked by miR-30b overexpression (Figure 4(b)). Consistently, exogenous expression of MRPL23-AS1 increased the transcriptional activity of β-catenin (Figure 4(c)) and the protein levels of MYH9, β-catenin, c-Myc and Cyclin D1 (Figure 4(d)), and these phenomena were also significantly blocked after ectopic expression of miR-30b (Figure 4(d)). Besides, MYH9 was frequently overexpressed in OS tissues as compared to adjacent normal tissues (Figure 4(e)), and it was strongly positively correlated with MRPL23-AS1 level (Figure 4(f)). Functionally, the enhanced cell proliferation and invasion were significantly abolished by miR-30b overexpression, MYH9 silencing or XAV-939 (a inhibitor of Wnt/β-catenin signaling) treatment (Figure 4(g,h)).

## Discussion

LncRNA has been identified as a fundamental regulator in cancer cell biology [12]. In the current study, we found a novel OS-associated lncRNA, MRPL23-AS1, which was significantly upregulated in OS tissues and cell lines. Further investigation revealed that MRPL23-AS1 was mainly located in the cytoplasm of OS cells, and could sponge miR-30b to elevate MYH9, activating the carcinogenic Wnt/β-catenin signaling (Figure 4(i)). However, a very recent study showed that MRPL23-AS1 was a nuclear lncRNA that acted as a scaffold for EZH2 to epigenetically silence E-cadherin, facilitating cystic carcinoma lung metastasis [13]. This paradoxical phenomenon may be explained by the temporal and spatial specific expression pattern of lncRNA [14]. Despite the subcellular localization of MRPL23-AS1 is different, the biological role is the same, is to promote cancer occurrence and development. The functional role and underlying mechanism of MRPL23-AS1 in other malignant tumors need to be further studied to evaluate its potential as a therapeutic target for pan-cancer.

The ceRNA network plays a critical role in human cell life, its dysregulation may lead to tumorigenesis and aggressive progression [15]. LncRNA is one of endogenous expressed non-coding RNA that has been well documented to be a ceRNA [16]. Through sponging miRNA, lncRNA is able to indirectly control gene expression, thus has important biological and pathological functions. However, the location of lncRNA is critical for its function, the prerequisite for lncRNA to adsorb miRNA is that it is in the cytoplasm [17]. In the present study, we performed qRT-PCR and FISH assays, and both results showed that MRPL23-AS1 was a cytoplasmic lncRNA in OS cells, hinting that MRPL23-AS1 might function by acting as a ceRNA. Subsequently, by a series of bioinformatics analyses and experiments, we identified that miR-30b was the downstream target of MRPL23-AS1. miR-30b belongs to miR-30 family, is a well-studied tumor suppressor in various human cancer types, including colorectal cancer [18], gastric cancer [19], medulloblastoma [20], hepatocellular carcinoma [21], esophageal squamous cell carcinoma [22] and OS [23]. Here, we also detected the level of miR-30b in OS tissues and normal tissues, found that it was dramatically decreased in OS tissues, and this phenomenon was also observed in OS cell lines. Moreover, ectopic expression of miR-30b could effectively block the enhanced malignant phenotype caused by MRPL23-AS1 overexpression, suggesting that miR-30b is a tumor-inhibiting factor in OS, and deregulation of MRPL23-AS1/miR-30b axis may be responsible for OS growth and spread.

MYH9 gene encodes a conventional non-muscle myosin that contains a myosin head-like domain which is involved in several important functions, including cell migration, adhesion, division, polarity and morphogenesis [24]. Up to now, many studies have shown that MYH9 is an oncogene in human cancer, including hepatocellular carcinoma [25], ovarian cancer [26], pancreatic cancer [27] and OS [28]. Patients with high MYH9 level had shorter survival time than those with low MYH9 level [29]. It can directly bind to β-catenin promoter and meanwhile recruited MLC9, β-actin and RNA polymerase II to facilitate β-catenin transcription, thereby activating carcinogenic Wnt/β-catenin pathway [13]. Here, we found that MYH9 is the downstream target of MRPL23-AS1/miR-30b axis, overexpression of MRPL23-AS1 activates Wnt/β-catenin pathway, and this effect was significantly abolished after miR-30b overexpression, MYH9 silencing or XAV939 treatment. These indicate that the regulatory axis of MRPL23-AS1/miR-30b/MYH9/Wnt/β-catenin is indeed present in OS cells. It is of great interesting to explore the causes of MRPL23-AS1 upregulation in OS tissues and cell lines, and whether MRPL23-AS1 is reversely regulated by β-catenin warrants further investigation.

## Conclusion

Our data demonstrate that MRPL23-AS1 is a novel driver of OS tumorigenesis and progression, providing a new insight into the pathogenesis of OS as well as a promising target for OS clinical treatment.

## Supplementary Material

Supplemental Material

## Highlights

1. MRPL23-AS1 is frequently overexpressed in OS and linked to poor prognosis.
2. MRPL23-AS1 promotes OS cell proliferation and invasion both *in vitro* and *in vivo*.
3. MRPL23-AS1 acts as a sponge of miR-30b.
4. MRPL23-AS1 regulates the miR-30b/MYH9/Wnt/β-catenin axis
