# Supplementary figures and images for: Long non-coding RNA (LncRNA) MRPL23-AS1 promotes tumor progression and carcinogenesis in osteosarcoma by activating Wnt/β-catenin signaling via inhibiting microRNA miR-30b and upregulating myosin heavy chain 9 (MYH9)
Source: Bioengineered. 2020 Dec 28;12(1):162–71. doi: 10.1080/21655979.2020.1863014 (PMC8806232; doi:10.1080/21655979.2020.1863014)

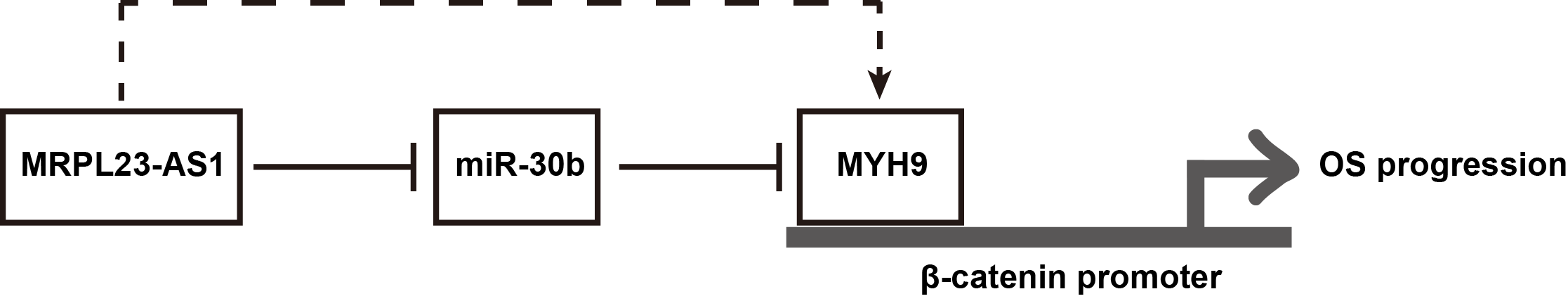

Supplement: Supplemental Material [file KBIE_A_1863014_SM8684.zip › supplement/Graphical Abstract.tif]
